# Novel Risk Factors for Uveal Melanoma in Adolescent and Young Adult Patients: A Comprehensive Case–Control Analysis

**DOI:** 10.1016/j.xops.2024.100687

**Published:** 2024-12-24

**Authors:** Arina Nisanova, Susanna S. Park, Aana Amin, Carly Zako, Machelle D. Wilson, Jessica Scholey, Armin R. Afshar, Tony Tsai, Devron H. Char, Kavita K. Mishra

**Affiliations:** 1 Department of Radiation Oncology, University of California San Francisco, San Francisco, California; 2 Department of Ophthalmology and Vision Science, University of California Davis, Sacramento, California; 3 Public Health Sciences, Division of Biostatistics, School of Medicine, University of California Davis, Sacramento, California; 4 Ocular Oncology Service, Department of Ophthalmology, University of California San Francisco, San Francisco, California; 5 Retinal Consultants Medical Group, Sacramento, California; 6 Tumori Foundation, San Francisco, California

**Keywords:** Uveal melanoma, Ocular melanoma, Adolescents and young adults, Risk factors, Proton beam radiation therapy

## Abstract

**Purpose:**

To identify risk factors associated with uveal melanoma (UM) in adolescents and young adults (AYAs).

**Design:**

A retrospective case–control study.

**Participants:**

Two hundred forty-seven UM patients aged 13 to 45 treated with proton beam radiation therapy and 401 age- and sex-matched controls at a tertiary academic center.

**Methods:**

We obtained demographic and genetic data, environmental exposures, and social, medical, and ocular history via retrospective chart review and phone follow-up.

**Main Outcome Measures:**

The main outcome measures included the prevalence and odds ratios (ORs) of the investigated risk factors in UM patients compared with controls.

**Results:**

The median age of UM diagnosis was 38 years (range: 13–45 years); the median follow-up was 102 months (range: 3–329 months). Identified novel risk factors for UM included family history of cutaneous melanoma (OR = 3.06, *P* = 0.002), Ashkenazi Jewish ancestry (2.98, *P* = 0.02), prior eye trauma (2.94, *P* = 0.01), secondhand cigarette smoke exposure (2.39, *P* < 0.001), and previous head and neck surgery (1.81, *P* = 0.007). Some known risk factors identified include choroidal nevi (11.39, *P* < 0.001), light eye color (4.69, *P* < 0.001), White race (4.63, *P* < 0.001), outdoor sunlight exposure (4.20, *P* < 0.001), recent pregnancy (4.0, *P* = 0.002), occupational (2.39, *P* = 0.003) and toxic chemical (2.27, *P* = 0.03) exposures, family history of any cancer (2.16, *P* < 0.001), lack of ultraviolet-blocking eyewear use (2.13, *P* = 0.01), indoor tanning (2.10, *P* = 0.03), and propensity to sunburn (1.89, *P* < 0.05). The prevalence of oculodermal melanocytosis (*P* = 0.03) and family history of UM (*P* < 0.001) were significantly greater in UM patients than in controls. Uveal melanoma T-categories were as follows: 39% T1, 37% T2, 19% T3, and 5% T4. Gene expression profiling was available in 64 patients and showed 59% class 1A, 19% class 1B, and 22% class 2 tumors. Thirteen patients underwent genetic screening; identified germline mutations included *CDH1, NF1,* and *PALB2.* The estimated 10-year metastasis-free progression rate and overall survival were 80% and 81%, respectively.

**Conclusions:**

This study identified several novel risk factors for UM in AYAs and confirmed select established risk factors seen in UM patients of all ages. To the best of our knowledge, this is the first explicit and comprehensive investigation of risk factors among a younger cohort and may help further elucidate UM pathogenesis.

**Financial Disclosure(s):**

Proprietary or commercial disclosure may be found in the Footnotes and Disclosures at the end of this article.

Uveal melanoma (UM) is the most common primary intraocular malignancy in adults. The yearly incidence is 5 to 6 per million.^1^ The peak age of UM diagnosis falls in the seventh decade of life, but UM has been diagnosed in all age groups.^2^ The pathophysiology of the disease is not completely understood. Multiple genetic abnormalities have been implicated in UM, including activating mutations of *GNAQ* and *GNA11* genes^3^^,^^4^ and abnormalities in chromosomes 1, 3, 6, 8, and 16.^5^ Despite the advancements in ocular therapies resulting in 90% to 95% of primary tumor control depending on modality, the 10-year survival rate has remained unchanged in the past several decades, with up to half of UM patients developing metastatic disease, which is typically fatal.^1^

Some established risk factors for UM include Northern European descent, light eye and light skin color,^6^^,^^7^ oculodermal melanocytosis (ODM),^8^ choroidal nevi,^9^ and cutaneous nevi.^7^ Several genetic factors may predispose to UM, including breast cancer gene (BRCA) associated protein-1 (BAP1) tumor syndrome,^10^ neurofibromatosis type I,^11^ and the familial atypical mole-melanoma syndrome.^12^ Uveal melanoma is less common in younger patients, and in earlier studies, pediatric and adolescent cohorts have shown better survival and lower metastatic disease rates compared with older adults.^13^, ^14^, ^15^ The risk factors for UM in younger patients have not been explicitly delineated previously. Some genetic and phenotypic features are likely applicable to the younger population; however, to our knowledge, there has not been a dedicated comprehensive analysis of risk factors for UM in younger patients. Increasingly, there is awareness that younger-aged cancer patients may have variable experiences and outcomes,^16^ with reports varying from cutoffs of 40 to 50 years of age for “young adults” in different cancer studies.^17^ Pica et al^16^ compared outcomes in UM patients treated with proton beam radiation therapy (PBRT) between older adults and adolescents and young adults (AYAs), defined as an age group between 15 and 39. Singh et al^2^ further reported that the incidence of UM ranges from 0.2 per million for ages 10 to 14 to 6.3 in adults aged 40 to 44. The incidence increases substantially for patients aged 50 to 55 to 17 per million and increases until reaching its peak between 70 and 74 years of age (42.3 per million).^2^ For these reasons, in our study, we included the AYA population between the ages of 13 and 45. Focused research into the potential medical, genetic, and environmental risk factors for UM in younger patients may help us understand the pathogenesis in this population. We conducted a cross-sectional case–control investigation of sociodemographic and past medical history of younger patients (13–45 years old) diagnosed with UM to elucidate potential novel risk factors associated with UM.

## Methods

This cross-sectional case–control study included 247 patients with UM and 401 age- and sex-matched controls. The study was conducted in accordance with the tenets of the Declaration of Helsinki and the Health Insurance Portability and Accountability Act. The institutional review board approved the study protocol at the University of California, Davis and the University of California, San Francisco.

### Patient Population

Uveal melanoma patients were identified using the prospectively maintained database of eyes treated with PBRT at University of California, San Francisco (n = 2558). The study inclusion criteria included UM diagnosed in AYAs, defined as ages 13 to 45, and treated with primary PBRT for nonmetastatic UM between 1994 and 2020. This identified 247 AYA patients with UM for our study analysis.

The control cohort was recruited from patients who received optometric care at the University of California, Davis. It included 401 age- and sex-matched patients aged 13 to 45 years old at the time of chart abstraction who had a normal optometric eye examination, permitting the presence of a refractive error, choroidal nevi, and ODM. Patients with other ophthalmic pathologies were excluded from the control cohort to mitigate potential bias that may arise from control patients receiving frequent ophthalmic screening for other ocular diseases. Additionally, we did not exclude control patients with choroidal nevi or ODM since these are known risk factors for UM. By including these diagnoses in the control group, we can compare the prevalence and odds ratios (ORs) of these features in the UM versus the control population and assess how these features affect the risk of developing UM in AYA patients.

The control cohort size (n = 401) was calculated based on the established prevalence of established risk factors, including ODM, light eye, and skin color. Although most risk factors could be detected with fewer controls, approximately 1.62 controls per UM patient were needed to detect a difference in ODM prevalence with 80% power at the 0.05 significance level. Uveal melanoma patients were age- and sex-matched to controls with a 1.62 ratio. Patients were randomly identified through an electronic medical record defined by age, sex, and availability of optometric care. The exclusion criteria for control patients included (1) known or discovered history of ophthalmic conditions, such as diabetic retinopathy, glaucoma, or any other ophthalmic conditions and (2) ophthalmic diagnoses besides refractive error, choroidal nevi, and ODM.

### Data Collection and Survey

Between June 2023 and May 2024, we conducted a retrospective chart review of patients' demographic information, environmental exposures, and social and medical history utilizing electronic medical record and supplemented data collection with a follow-up phone call to obtain any data that were not available in the patient's chart. All UM participants signed an informed consent before enrollment. For control patients, written consent was not required per the institutional review board–approved waiver of participants' authorization for retrospective chart review. However, both UM and control participants provided verbal consent prior to completing the phone questionnaire.

Demographic and host factors (Table S1, available at www.ophthalmologyscience.org) included age at diagnosis for UM patients or age at the time of chart abstraction for control patients, race, Ashkenazi Jewish ancestry (binary: yes or no), and eye color classified as light (blue, green, or gray) or dark (brown) and propensity to sunburn (yes/no). We recorded sunlight and ultraviolet (UV)-related factors, including subjectively reported average weekly time spent outdoors during daylight (rated as <3 hours, 3–10, 11–20, or >20 hours per week), regular use of UV-blocking eyewear (sunglasses, prescription glasses, contact lenses, or none), and tanning bed use (yes/no), followed by frequency and ages during which the exposure occurred if positive. Additionally, we collected history of being an airplane pilot (yes/no) followed by the route and the number of flights completed if positive.

We also recorded social and environmental history (Table S1) and primary occupation. We also obtained a history of heavy alcohol use (yes/no), where heavy drinking was defined as drinking ≥5 drinks on any day or ≥15 drinks per week for men and >4 drinks per day or 8 drinks per week for women.^18^ Tobacco use (yes/no) was also recorded, including the type of tobacco products used (cigarettes, cigars, vapes, or chewing tobacco), the pack-year history for cigarette smokers, and subjectively reported or medically documented significant secondhand cigarette smoke exposure (yes/no). We also obtained any documented or patient-reported exposure to chemicals or retinoid therapy use (oral or topical).

The survey of prior medical history (Table S1) included binary variables (yes/no) of past head and neck surgical history, prior cancer diagnoses, and family history of cancer. If the patients answered yes to any of these questions, further details were recorded, including the number and type of prior diagnosis and head and neck surgeries; type of cancer diagnosis, modality of treatment, and outcome; the number of family members affected by cancer, their relationship to the patient, and the type of cancer they had. The survey of ocular history included binary variables (yes or no) of prior eye trauma, choroidal nevi, ODM, and laterality if history was positive for any of these factors. Eye trauma was defined as a history of blunt or penetrating trauma to the eye as reported by the patient in the phone interview or as documented in the electronic medical record. For female patients, we also recorded recent pregnancy, defined as pregnancy at the time of or within a year preceding the UM diagnosis for UM patients and at the time of or within a year preceding the chart abstraction for control patients. We also recorded whether a patient had ever been pregnant before, outside of the 1-year UM diagnosis window.

For UM patients, we obtained the tumor history, including the first date of PBRT, tumor laterality, stage, dimensions, and location in the eye. Fine needle aspiration biopsy details, including tumor cytology and gene expression profile (GEP) class and the next-generation sequencing (NGS) results, were recorded when available. Additionally, we collected follow-up history, including the name of the treating ocular or medical oncologist, the date of the last follow-up with the provider, and any significant history after PBRT, including the presence of distant metastasis from UM and metastatic biopsy details, other primary cancer diagnoses after UM, and the death date when applicable.

### Selection of the Surveyed Risk Factors

We conducted a literature search on the risk factors implicated in UM. In addition to surveying more established risk factors that were discussed in the introduction, we also encountered several reports on potential associations of other factors with UM. Namely, Ashkenazi Jewish was identified as a variable of interest due to a significantly higher incidence of UM in Israel among Jews of Ashkenazi compared with Middle Eastern, North African, and Asian ancestries reported by Iscovich et al.^19^^,^^20^ Retinoid use was analyzed as follow-up on a case series of younger patients with UM and history of retinoid use recently described by our group.^21^ The evidence regarding pregnancy is equivocal^22^ and thus was included in our study. Several airline pilots were encountered by our group in clinical practice; although this occupation has an established risk for cutaneous melanoma,^23^ there is no evidence regarding UM. There are several reports posing an association between UM and eye trauma;^24^, ^25^, ^26^, ^27^ however, this association has never been explored in a case–control study and was therefore included in our study. History of prior head and neck surgeries was surveyed as part of the general medical history assessment.

### Statistical Analysis

We used Microsoft Excel (version 16.68, Microsoft Corp), GraphPad Prism 10 (version 9.5.0, GraphPad Software, Inc), and SAS software (version 9.4, SAS Inc) to analyze the data and generate descriptive statistics. Comparison of prevalence was computed using the chi-square analysis and logistic regression to estimate ORs, and the findings were considered significant if the *P* value was <0.05. We conducted a complete case analysis. Subjects with missing values were dropped from analyses that included those variables. Survival probabilities were calculated using the Kaplan-Meier method. The results were considered statistically significant if the 95% confidence interval did not include the value of one.

## Results

Of 2558 eyes treated with PBRT at University of California, San Francisco, 247 eyes (247 patients; 10%) met the inclusion criteria by age and were included in the study. We also reviewed 589 charts of potential control patients, of which 188 were excluded due to corneal and retinal pathology, congenital eye disorders, inflammatory ocular conditions, and history of retinoblastoma. The final control cohort included 401 patients.

Of 247 UM patients, 120 (49%) were male. Most UM patients were White (n = 203, 82%), 22 (9%) Hispanic or Latino, 8 (3%) Asian, 5 (2%) Middle Eastern/North African, 2 (1%) Black/African American, and 1 (0.5%) Native American. Six (2.5%) patients reported mixed race. The median age at UM diagnosis was 38 years old (range: 13–45 years). The median follow-up time since UM diagnosis was 102 months (range: 3–329 months). The median age of control patients was 38 years (range: 13–45 years). Of 401 control patients, 195 (49%) were male. Most control patients were White (n = 200, 50%), 68 (17%) were Asian, 68 (17%) were Hispanic or Latino, 31 (8%) were Black or African American, 7 (2%) were Middle Eastern/North African, and 3 (1%) were Native American. The race of 24 (5%) of the control patients was unknown. Comparisons of risk factor prevalence and ORs are summarized in Table 2, Table 3, Table 4 in order of decreasing ORs by the risk factor group.

**Table 2:** Demographic, Host, Sunlight, and Artificial UV Light Exposures

Risk Factor	UM (n, %)	Control (n, %)	*P* Value	Odds Ratio	95% CI
Eye color					
Light	61 (69%)	46 (32%)	<0.001	4.69	2.67, 8.33
Dark	28 (31%)	99 (68%)		0.21	0.12, 0.37
Race: White					
Yes	203 (82%)	200 (50%)	<0.001	4.63	3.19, 6.78
No	44 (18%)	201 (50%)		0.22	0.15, 0.31
Weekly time outdoors (>20 hours)					
Yes	46 (53%)	23 (21%)	<0.001	4.20	2.23, 8.00
No	41 (47%)	86 (79%)		0.24	0.13, 0.45
Weekly time outdoors (>10 hours)					
Yes	55 (63%)	41 (38%)	<0.001	2.85	1.56, 5.18
No	32 (37%)	68 (62%)		0.35	0.19, 0.64
Ashkenazi Jewish ancestry					
Yes	13 (9%)	7 (3%)	0.02	2.98	1.21, 7.67
No	134 (91%)	215 (97%)		0.33	0.13, 0.83
Race: Hispanic or Latino					
Yes	22 (9%)	68 (17%)	0.003	0.47	0.29, 0.77
No	225 (91%)	333 (83%)		2.13	1.30, 3.48
UV-blocking eyewear use					
Yes	58 (73%)	230 (85%)	0.01	0.47	0.26, 0.84
No	22 (28%)	41 (15%)		2.13	1.19, 3.78
Indoor tanning					
Yes	22 (19%)	16 (10%)	0.03	2.10	1.06, 4.11
No	91 (81%)	139 (90%)		0.48	0.24, 0.94
Proneness to sunburn					
Yes	71 (79%)	73 (66%)	<0.05	1.89	1.02, 3.56
No	19 (21%)	37 (34%)		0.54	0.53, 0.98

CI = confidence interval; UM = uveal melanoma; UV = ultraviolet.

**Table 3:** Social and Environmental Factors

Risk Factor	UM (n, %)	Control (n, %)	*P* Value	Odds Ratio	95% CI
Secondhand cigarette smoke exposure					
Yes	43 (42%)	58 (23%)	<0.001	2.39	1.44, 3.95
No	59 (58%)	190 (77%)		0.42	0.25, 0.70
High-risk occupation∗					
Yes	29 (15%)	21 (7%)	0.003	2.39	1.33, 4.29
No	159 (85%)	275 (93%)		0.42	0.23, 0.75
Chemical exposure†					
Yes	25 (16%)	10 (8%)	0.03	2.27	1.05, 4.70
No	131 (84%)	119 (92%)		0.44	0.21, 0.95
Smoking pack-years					
Mean (SD)	7.9 (7.7)	5.1 (6.4)	0.04		
Median (interquartile range)	6 (2, 11)	3 (0.5, 7)			
Range	0.05, 40	0.025, 30			
Heavy alcohol use					
Yes	12 (6%)	24 (6%)	1	1.0	0.50, 1.99
No	176 (94%)	351 (94%)		1.0	0.50, 1.99
History of smoking					
Yes	61 (32%)	99 (26%)	0.11	1.37	0.93, 1.99
No	127 (68%)	282 (74%)		0.73	0.50, 1.07
Retinoid therapy					
Yes	20 (20%)	57 (23%)	0.53	0.83	0.47, 1.45
No	81 (80%)	192 (77%)		1.20	0.69, 2.11
Pilot					
Yes	2 (1%)	1 (0.3%)	0.29	3.37	0.3, 49.0
No	179 (99%)	302 (99.7%)		0.30	0.02, 2.57

CI = confidence interval; SD = standard deviation; UM = uveal melanoma.

∗ High-risk occupation was defined as occupations, including welders, dry cleaners, occupational cooks, military and construction workers, firefighters, mechanics, and farmers, who are at an increased risk of exposure to carcinogenic substances, including per- and polyfluorinated substances, organic solvents, and pesticides.

† Chemical exposure was defined as exposure to asbestos, pesticides, organic solvents, phenols, and formaldehyde.

**Table 4:** Medical and Clinical Risk Factors

Risk Factor	UM (n, %)	Control (n, %)	*P* Value	Odds Ratio	95% CI
Choroidal nevi					
Yes	23 (13%)	5 (1%)	<0.001	11.39	4.28, 27.9
No	153 (87%)	379 (99%)		0.09	0.04, 0.23
Family history of cutaneous melanoma					
Yes	21 (12%)	15 (4%)	0.002	3.06	1.57, 5.97
No	158 (88%)	345 (96%)		0.33	0.17, 0.64
Recent pregnancy					
Yes	13 (13%)	7 (4%)	0.002	4.0	1.64, 10.20
No	85 (87%)	183 (96%)		0.25	0.10, 0.61
Prior eye trauma∗					
Yes	12 (7%)	9 (2%)	0.01	2.94	1.23, 7.25
No	169 (93%)	372 (98%)		0.34	0.14, 0.80
Family history of any cancer					
Yes	122 (68%)	179 (50%)	<0.001	2.16	1.49, 3.17
No	57 (32%)	181 (50%)		0.46	0.32, 0.67
Prior head and neck surgery					
Yes	46 (24%)	57 (15%)	0.007	1.81	1.18, 2.82
No	145 (76%)	326 (85%)		0.52	0.35, 0.85
Family history of ocular melanoma					
Yes	3 (1%)	0 (0%)	0.01	—	—
No	176 (99%)	360 (100%)		—	—
Oculodermal melanocytosis					
Yes	2 (1%)	0 (0%)	0.03	—	—
No	178 (99%)	383 (100%)		—	—
Prior cancer diagnosis					
Yes	13 (7%)	17 (4%)	0.22	1.59	0.79, 3.25
No	178 (93%)	369 (96%)		0.63	0.31, 1.27
Pregnancy before diagnosis					
Yes	50 (49%)	98 (53%)	0.52	0.85	0.52, 1.39
No	52 (51%)	87 (47%)		1.17	0.72, 1.91

CI = confidence interval; UM = uveal melanoma.

∗ Eye trauma was defined as a history of blunt and penetrating eye injury to the uveal melanoma eye.

Past medical, family, and follow-up history, as well as alcohol, tobacco use, and occupation, were mostly available in the medical records. Assessment of host factors, including eye color and ancestry, behavioral and sun-related factors, and chemical exposures, most commonly necessitated phone follow-up. Seventy-seven (39%) of 197 UM patients who were alive at the time of the study completed an additional follow-up phone survey. For this additional phone call, we were unable to reach 119 UM patients, of whom 5 resided outside of the United States (US) and 1 declined to participate. Of 401 control patients, 94 (23%) completed the phone survey, 80 (20%) declined to participate, and 227 (57%) did not answer the phone.

### Demographic, Host, UV-Related Factors

Light eye color (OR = 4.69, *P* < 0.001), White race (OR = 4.63, *P* < 0.001), and Ashkenazi Jewish ancestry (OR = 2.98, *P* = 0.02) showed a strong association with developing UM. In our study, patients of this ancestry represented 7% of the entire UM cohort (or 9% of 167 confirmed cases) compared with 1.5% to 2% of the US population.^28^ Hispanic or Latino ethnicity was protective against UM (OR = 0.47, *P* = 0.003). Greater than 20 (OR = 4.20, *P* < 0.001) or 10 hours (OR = 2.85, *P* < 0.001) of weekly outdoor time conferred a significantly greater risk for UM. The risk of UM appeared to be time-dependent—the risk is greater for those with >20 hours of weekly outdoor activity when compared with those with 10 hours of weekly outdoor activity. Additionally, the lack of UV-blocking eyewear use (OR = 2.13, *P* = 0.01), history of indoor tanning (OR = 2.10, *P* = 0.03), and propensity to sunburn (OR = 1.89, *P* < 0.05) were associated with a significantly increased risk of UM. History of working as a pilot (OR = 3.37, *P* = 0.29) was not statistically significant.

### Social and Environmental Factors

Exposure to secondhand cigarette smoke (OR = 2.39, *P* < 0.001) showed significant associations with UM. In our study, we identified several “high-risk” occupations based on prior reports linking UM to occupational welding,^7^^,^^29^^,^^30^ dry cleaning jobs,^31^ occupational cooking,^32^ and construction.^31^ Additionally, we included in our “high-risk” occupation military workers, firefighters, mechanics, and farmers who are at an increased risk of exposure to carcinogenic substances, including per- and polyfluorinated substances, organic solvents, and pesticides.^30^^,^^33^ Exposure to toxic chemicals was defined as exposure to asbestos, pesticides, organic solvents, phenols, and formaldehyde due to their implications in UM and other malignancies.^30^^,^^31^^,^^34^ High-risk occupation (OR = 2.39, *P* = 0.003) and chemical exposure (OR = 2.27, *P* = 0.03), as defined in our study, showed an increased risk for UM. History of smoking (OR = 1.37, *P* = 0.11), heavy alcohol use (OR = 1.54, *P* = 0.24), and prior retinoid therapy (OR = 0.83, *P* = 0.53) showed no significant associations with UM. However, the average pack-year smoking history was significantly greater in UM patients compared with controls (7.9 vs. 5.1 pack-years, *P* = 0.04).

### Medical and Clinical Risk Factors

Table 4 summarizes medical and clinical risk factors evaluated for possible association with UM. Positive family history of any cancer was significantly greater (OR = 2.16; *P* < 0.001) in the UM cohort (122 of 179 patients, 68%) compared with controls (179 of 360, 50%). Three (1%) UM patients reported a family history of ocular melanoma, which was significantly higher than none reported in the control group (*P* = 0.01). In addition, 21 UM patients (9%) had a family history of cutaneous melanoma (OR = 3.06; *P* = 0.002)—14 (67%) in the first-degree relatives and 7 (23%) in the second-degree relatives.

The presence of pre-existing choroidal nevi in either eye (OR = 11.39, *P* < 0.001) was associated with UM risk. Twenty-three (9%) UM patients had preexisting choroidal nevi, of whom 21 were in the UM eye and 2 in the contralateral eye, versus 5 of 384 (1%) patients in the control group. Transformation of preexisting choroidal nevi to UM was seen in 21 of 179 UM patients (12%). Notably, 2 of our patients had preexisting choroidal nevi that showed growth and malignant transformation during pregnancy, and another patient developed a nevus while pregnant, which transformed into UM 3 years later. History of ODM was noted in 2 of 180 (1%) patients with UM and none among the controls (*P* = 0.03).

Other medical and clinical risk factors associated with UM include prior trauma in the UM eye (OR = 2.94; *P* = 0.01) and history of head and neck surgeries (OR = 1.81; *P* = 0.007). Prior eye trauma was reported by 15 UM patients (7% of 181), 12 of which affected the UM eye, including 2 reports of bilateral eye trauma. Two patients had trauma in the contralateral eyes, and 1 patient could not recall which eye was affected; thus, only 12 UM eyes were included in the analysis. Forty-six UM patients (24% of 191) had a prior history of head and neck surgeries, of which tonsillectomies and adenoidectomies (22, 48%) and wisdom teeth extractions (17, 37%) were most frequently reported. Other surgeries included brain surgery for intracranial hematoma, cervical spine surgery, cranial and maxillofacial reconstruction secondary to trauma or overbite correction, neck lymph node dissection, thyroidectomy, septo/rhinoplasty, sinus surgery, and skin cancer resection.

Among female UM patients (n = 127), 13 (13% of 98) were pregnant at the time of or within a year preceding UM diagnosis, and 50 (49% of 102) patients in total had ever been pregnant before the UM diagnosis. Recent pregnancy (OR = 4.0, *P* = 0.002) showed significant associations with UM, while ever pregnancy preceding the UM diagnosis showed no association (OR = 0.85, *P* = 0.52).

### Features of UM

Tumor features and genetic testing details are summarized in Table 5. Uveal melanoma affected the left eye in 54% of cases (n = 134). The median tumor height was 4.4 mm (range: 0.8–15.8), the median largest tumor diameter was 10.5 mm (range: 2.3–25.1), and the median distance to disc/fovea was 3.1/1.9 mm. Based on the largest tumor diameter and thickness, 97 (39%) UM were category T1, 92 (37%) were category T2, 47 (19%) were T3, and 11 (5%) eyes had T4 tumors. Most UMs were confined to the choroid (87%), 12% involved the ciliary body, and 1% were confined to the iris only. Fine needle aspiration biopsy results were available for 119 (48%) patients. Of accessible cytology results (n = 98), 68 (69.4% of 98) were spindle cell/low-grade tumors, 21 (21.4%) had epithelioid features, and 9 (9.2%) were mixed-cell type. The GEP results were available for 64 patients who had a fine needle aspiration biopsy (54% of 119 patients) and showed 38 (59%) class 1A tumors, 12 (19%) class 1B, and 14 (22%) class 2 tumors. Next-generation sequencing results were available for 13 (5%) patients and identified the following mutations: *GNAQ* (n = 6), *SF3B1* (n = 5), *EIF1AX* (n = 3), *GNA11* (n = 3), *APC* (n = 1), *FBXW7* (n = 1), *PABL2* (n = 1), *NF1* (n = 1), and *CDH1* (n = 1).

**Table 5:** Tumor Characteristics and Genetic Screening Results

Patient and Tumor Characteristics	UM (n = 247)
Median age at diagnosis (range)	38 (13–45)
Sex	
Female	127 (51%)
Male	120 (49%)
Laterality	
Right	113 (46%)
Left	134 (54%)
Primary tumor location	
Choroid	215 (87%)
Ciliary body	30 (12%)
Iris only	2 (1%)
Tumor dimensions (mm)	Median (range)
Height	4.4 (0.8, 15.8)
Largest basal diameter	10.5 (2.3, 25.1)
Tumor T-category	n (%)
T1	97 (39%)
T2	92 (37%)
T3	47 (19%)
T4	11 (5%)
Fine needle aspiration biopsy	n = 119 (48%)
Tumor cytology based on biopsy	n = 98
Spindle cell/low-grade	68 (69.4%)
Epithelioid cell	21 (21.4%)
Mixed-cell type	9 (9.2%)
Gene expression profile class	n = 64
1A	38 (59%)
1B	12 (19%)
2	14 (22%)
Next-generation sequencing	n = 13
GNAQ (somatic)	6
SF3B1 (somatic)	5
GNA11 (somatic)	3
EIF1AX (somatic)	3
APC (somatic)	1
FBXW7 (somatic)	1
CDH1 (germline)	1
PABL2 (germline)	1
NF1 (germline)	1

UM = uveal melanoma.

The estimate of overall survival was 81% (95% confidence interval: 74%–86%) at 10 years. The estimate of metastasis-free survival was 80% at 10 years (95% confidence interval: 74%–86%). With the 102-month median follow-up, 50 (20%) patients had deceased, of which 45 deaths (90%) were related to metastatic disease as per the last available follow-up. Among patients with distant metastasis, GEP was available for 14 patients and showed 1 (7%) GEP class 1A tumor, 3 (21.5%) class 1B tumors, and 10 (71.5%) class 2 tumors.

### Other Primary Cancers

We found that 37 (15%) UM patients had other primary cancers (OPCs) besides UM, of which 13 (35%) had an OPC diagnosed before UM diagnosis, and 25 (68%) developed an OPC after UM diagnosis. One patient (3%) had 2 other primary malignancies, both before and after UM diagnosis. History of OPC before UM diagnosis did not significantly increase the risk for UM (OR = 1.56, *P* = 0.22). Most frequent OPCs included nonmelanoma skin cancer (n = 11, 31%), cutaneous melanoma (7, 19%), breast cancer (4, 11%), cancers of the blood, bone marrow or lymph (3, 8%), and prostate cancer (2, 6%). Other identified OPCs included cervical, ovarian, and endometrial cancer; renal cell carcinoma; rhabdomyosarcoma, thyroid, oral, throat, stomach, and testicular cancers.

Pathogenic germline mutations suggestive of a hereditary cancer syndrome were identified in 2 patients on NGS. One patient developed endometrial cancer after her UM diagnosis. Her endometrial tumor harbored a somatic *MSH6* mutation without a detectable germline mutation in any of the mismatch repair genes; however, she was found to have a pathogenic germline *CDH1* mutation (c.715G > A (p.Gly239Arg) on NGS. The second patient was found to have 2 pathogenic germline mutations in *NF1* and *PALB2* genes. This patient developed distant metastasis in the liver from UM 9 years (112 months) after her UM diagnosis. She was alive at the time of the last follow-up, 9 months after metastatic UM diagnosis, and did not have any evidence of OPCs at the time of the last available follow-up. None of the NGS-tested patients (n = 13) had a *BAP1* germline mutation.

## Discussion

Although UM is the most common primary intraocular cancer in adults, it remains a relatively rare malignancy. Prior studies have identified various risk factors for UM, including older age, White race, light skin, and eye color. These known risk factors for UM suggest an important role of UV light exposure in the pathogenesis of UM. The risk factors specific to UM in AYAs have not been studied previously because the incidence of UM in a younger population is especially rare. Prior studies have shown a better prognosis for UM in young patients (pediatric and adolescents) than in older adults.^13^, ^14^, ^15^^,^^35^^,^^36^ In our retrospective cross-sectional study, risk factors for UM in AYAs were identified using age- and sex-matched healthy patients as controls. Our study identified several novel risk factors for UM in AYAs and confirmed many of the previously identified risk factors associated with all UM patients. Each class of risk factors identified in our study is discussed in more detail below.

### Family History of Cutaneous Melanoma

A strong family history of cancer is an established risk factor for UM^37^ and was also identified as a significant risk factor in our younger cohort. Family history of cutaneous and UM, specifically, posed a significantly higher risk for developing UM in our study. Uveal melanoma has been observed in families with a history of cutaneous melanoma and familial atypical multiple mole melanoma syndrome.^12^^,^^38^ A previous case–control study described 6 cases of UM in association with cutaneous melanoma.^12^^,^^39^ Our study is the first to implicate it as a statistically significant risk factor for UM.

### Ashkenazi Jewish Ancestry

Ashkenazi Jewish ancestry (Jews of Eastern European descent) was associated with a greater risk of developing UM in our younger patients. Ashkenazi Jews have an increased susceptibility to multiple cancers,^40^ and UM in Israel is reportedly diagnosed 3 to 4 times more frequently among Jews of European than Asian or African descent.^19^^,^^20^ Ashkenazi Jews typically have a phenotype consistent with light skin and eyes. It is unclear if phenotypical features alone explain the difference in UM prevalence or whether genetic factors may also play a role. One potential limitation is referral pattern bias for Ashkenazi Jewish patients in our region, though, upon further review, 13 patients were referred for PBRT by 6 different ocular oncologists across the region. To our knowledge, this is the first case–control study to document Ashkenazi Jewish ancestry as a significant risk factor for UM compared with the general US population, but further studies are needed to investigate this and determine whether the risk applies to all UM patients.

### Eye Trauma

Rare case reports posed a possible association between trauma and UM.^24^, ^25^, ^26^, ^27^ To our knowledge, this is the first case–control study to implicate eye trauma as a risk factor for UM. There was no clinical difference noted in the surveillance pattern for patients with prior eye trauma before they were diagnosed with UM. Considering an increased chance of developing UM observed in our UM patients, obtaining a comprehensive ocular history is warranted at routine eye examinations and initial ocular oncology evaluations.

### Secondhand Cigarette Smoke Exposure and Behavioral Factors

Certain lifestyle behaviors can predispose to oncologic disease,^41^^,^^42^ but their possible role in UM pathogenesis is equivocal. Secondhand cigarette smoke exposure carries an increased risk for multiple oncologic diseases,^43^ but our study is the first to implicate it as a significant risk factor for UM. Additionally, our findings suggest there may be a dose-dependent effect of tobacco smoking, as UM diagnosis was more likely in patients with greater pack-year smoking history.

### Head and Neck Surgeries

We also observed an increased risk of UM with a prior history of head and neck surgeries. An increased prevalence of non-UM-related surgical interventions has been reported among UM patients with OPCs,^44^ but we found no prior studies linking head and neck surgeries to UM. Prior studies reported a modestly increased risk of cancer among patients with prior tonsillectomies and adenoidectomies.^45^^,^^46^ However, this increased risk with tonsillectomies and adenoidectomies does not fully explain the increased risk of UM, given the additional head and neck surgeries reported in our UM patients. Additionally, patients with prior surgical history may have undergone more frequent health examinations, resulting in earlier UM detection. More evidence is needed to determine the role of prior nonocular surgical interventions in UM.

### Choroidal Nevi and Oculodermal Melanocytosis

Choroidal nevi are fairly common benign pigmented intraocular tumors that have a small potential for transformation into UM.^9^ History of choroidal nevi, in eyes both ipsilateral and contralateral to the tumor, conferred the strongest risk for UM in our study, although the known annual incidence of malignant transformation is estimated at 1 in 8845.^9^ Uveal melanoma arising from preexisting nevi (8%) in our cohort may be an underestimate as most of our UM patients likely did not have regular eye examinations prior to UM diagnosis. The prevalence of nevi noted in the control group (1.3%) may also be underestimated, considering the previously reported incidence of 5% to 8% among the US adults.^9^ This potential underestimate may have resulted in an overestimated OR for this risk factor in our study.

Oculodermal melanocytosis is a congenital benign hyperpigmentation of the uvea and periocular region,^47^ a known predisposing factor for primary UM and metastatic disease.^48^^,^^49^ Preexisting ODM was seen in 2 of our UM patients, neither with evidence of local recurrence nor distant metastasis at the time of the last follow-up.

### Recent Pregnancy

There is conflicting evidence for the association between pregnancy and UM in prior studies.^50^, ^51^, ^52^ To our knowledge, this is the first case–control study to examine the incidence of UM diagnosis with recent pregnancy specifically and implicate it as a risk factor. Prior pregnancy was not a risk factor for UM in our cohort, contrary to Zinkhan et al's findings.^50^ Accelerated UM growth in pregnancy has also been reported^53^ and also seen in 2 patients in our UM cohort. Closer surveillance of preexisting nevi or those developed during pregnancy may be warranted.

### Phenotypic Features

Phenotype consistent with light-colored eyes and skin is a generally accepted risk factor for UM.^54^, ^55^, ^56^ In our study, light iris color and White race conferred a strong risk for UM diagnosis in younger patients, while Hispanic/Latino ethnicity was protective against UM. These associations may indicate that exogenous UV light exposure may play a role in UM pathogenesis, even in young adults.

### Sunlight and Artificial Light Exposures

Our overall findings support the possible role of sunlight and artificial UV light in the pathogenesis of UM in young adults. The role of sunlight exposure and related factors in UM has been explored in multiple studies, but these studies have shown contradictory findings.^6^^,^^55^, ^56^, ^57^ Similar to some prior studies, our study associated UM risk with propensity to sunburn, increased sunlight exposure, lack of UV-blocking eyewear use, and indoor tanning.^6^^,^^7^^,^^55^, ^56^, ^57^, ^58^, ^59^ Although they have an established risk for cutaneous melanoma,^23^ they did not appear to have an increased risk of UM in our study.^7^^,^^29^^,^^55^, ^56^, ^57^, ^58^ Specific cohorts, such as pilots, were present in small numbers, and further study may be required for a specific subset of patients.

### Chemical and Occupational Exposures

In our study, history of employment in high-risk jobs and chemical exposures showed a higher risk of UM, consistent with previous reports.^29^, ^30^, ^31^, ^32^ Due to a limited number of patients with chemical or occupational exposures, we pooled a range of chemical exposures and occupations into combined variables. Although this approach may impact the accuracy of association with UM, the findings were significant and warrant further investigation.

### Retinoid Therapy

Retinoid therapy is associated with multiple adverse ocular effects.^60^ We recently described a series of younger UM patients with a recent history of topical isotretinoin and retinoid use.^21^ Although our present study did not show an association between UM and retinoid therapy, many of our patients were treated for UM in the 1990s and early 2000s before retinoid therapy became more popularized for cosmetic applications. Additionally, findings may be limited by the availability of records in the medical charts, as over-the-counter formulations may not have been included on the medication list. Further investigation in a cohort of more recently diagnosed patients may be warranted to elucidate the implications of retinoid use on ocular health and its potential role in UM.

### Other Primary Cancers

Uveal melanoma patients may also be at greater risk for hereditary cancer syndromes^61^ and OPCs.^62^ The incidence of multiple primary malignancies in our cohort (15%) was higher compared with those reported in the general cancer population (2.4%–8%).^63^ Two of our patients had documented pathogenic germline mutations: 1 in the *CDH1* gene and 1 in the *PABL2* and *NF1* genes. Germline mutations in these genes are commonly associated with hereditary malignancies.^61^ Thus, thorough characterization of the family cancer tree and genetic testing may be warranted in AYAs with newly diagnosed UMs to inform the necessity of screening for other malignancies.

### Outcomes

Our group has reported UM outcomes in younger UM patients,^62^ and a detailed analysis of long-term outcomes and survival is forthcoming. Uveal melanoma in pediatric patients seems to have better prognoses than the general UM population.^13^, ^14^, ^15^ The 10-year overall survival (81%) and metastasis-free survival (80%) estimates for our AYA cohort are higher than might be expected in the overall UM population (10-year overall survival: 55%, metastasis-free survival: 73%).^64^ The GEP and cytology results, available in 26% and 40% of the UM cohort, respectively, also support better prognosis of UM in AYA patients.^65^

### Limitations

The retrospective nature of this investigation is naturally a limitation of the study. The availability of certain sociodemographic and behavioral factors was limited without phone follow-up. The reporting of social and environmental exposures was based on the recall by patients and the availability of documented findings in medical charts. Thus, it was subject to both recall bias and dependent on the thoroughness of medical notes. Specifically, variables such as secondhand smoke exposure, sun exposure, and history of eye trauma may be prone to recall bias. Certain variables were limited to the phone survey if the data were not available in the medical chart; hence, missing data could have introduced bias into analysis. Presumably, this would be a similar issue for both case and control groups. The results may also be biased toward UM patients who remained alive if the sociodemographic information was not readily available in deceased patients' charts. Additionally, the results of this study are limited to patients who were treated with PBRT. While no obvious differences in risk factors have been noted between proton and other eye radiotherapy treatments in our region, there may be subtle underlying differences. We believe that our results raise questions that should be investigated further and may additionally prompt the inclusion of these questions in the initial ocular oncology or radiation oncology visits, thereby increasing the accuracy in subsequent studies.

### Strengths

Our study captured a relatively large cohort of younger-aged UM patients and included a thorough assessment of multiple implicated risk factors for UM. We identified several new risk factors associated with UM in this younger cohort. To the best of our knowledge, this is the first explicit, comprehensive investigation of risk factors among AYA patients with UM. The surveyed factors (Table S1) can also serve as a foundation for future work across institutions, registries, and patient-facing organizations to potentially glean more complete information for the special subset of AYAs.

In summary, the present study provides a comprehensive investigation of risk factors for UM among adolescent and young adult patients aged 13 to 45. We identified several novel risk factors for UM, including a family history of cutaneous melanoma, Ashkenazi Jewish ancestry, prior eye trauma, secondhand cigarette smoke exposure, and previous head and neck surgery. Additionally, we confirmed the prevalence of certain established risk factors among this younger population, including choroidal nevi, light eye color, White race, increased time spent outdoors, recent pregnancy, occupational and chemical exposures, family history of cancer, indoor tanning, lack of UV-blocking eyewear use, ODM, and familial UM. Our findings also support better prognosis in AYAs compared with older patients with UM based on overall survival, histopathologic, and genetic tumor profiles. Further investigation is needed to confirm the newly identified risk factors and determine their significance in the general UM population. Given multiple risk factors that have been identified in association with UM, our study highlights the importance of obtaining a complete medical, social, and family history as a risk assessment to evaluate all patients at risk for UM. This is a comprehensive risk factor study among adolescent and young adult UM patients, and the surveyed factors are foundational for future multi-institutional prognostic and pathogenesis work in UM patients.
