# Supplementary material for: Novel Risk Factors for Uveal Melanoma in Adolescent and Young Adult Patients: A Comprehensive Case–Control Analysis
Source: Ophthalmol Sci. 2024 Dec 24;5(4):100687. doi: 10.1016/j.xops.2024.100687 (PMC12002997; doi:10.1016/j.xops.2024.100687)
Supplement: Table S1 [file mmc1.pdf]

Table S1. Questionnaire of demographic, host, social and environmental, medical and clinical risk factors. All questions were surveyed in relation to the exposures that occurred before the uveal melanoma (UM) diagnosis for the UM cohort and before the time of chart abstraction for the control patients.

Question	Response options
Age	At diagnosis (UM)<br>At chart abstraction (controls)
Gender	Female<br>Male
Race	American Indian/Alaskan Native<br>Asian<br>Black/African American<br>Middle Eastern/North African<br>Hispanic/Latino<br>White (not Hispanic/Latino)<br>Mixed race
Ashkenazi Jewish ancestry	Yes<br>No
Patient's predominant eye color	Light (blue, green, or gray)<br>Dark (brown)
Is the patient prone to sunburns?	Yes<br>No
Average weekly time the patient spend outdoors in the sunlight?	Less than 3 hours<br>3 to 10 hours<br>10 to 20 hours<br>More than 20 hours
Did the patient regularly wear UV-blocking eyewear when in the sunlight?	Yes<br>Sunglasses<br>Contact lenses<br>Spectacles<br>No
Did the patient use tanning beds?	Yes, *if positive:*<br>Age and frequency of exposure<br>No
Did the patient ever worked as commercial airline pilot?	Yes<br>Number of years worked<br>Number of flights completed<br>Route of flights operated<br>No
High risk occupation?	Yes, if any of the following jobs:<br>Welder, dry cleaner, occupational cook, construction worker, military worker, firefighter, mechanic, farmer<br>No
History of heavy alcohol use?	Yes<br>No
History of tobacco use?	Yes, *if positive:*<br>Type of tobacco used?<br>Pack-years for cigarettes<br>Duration of tobacco use<br>No
History of significant secondhand cigarette smoke exposure?	Yes<br>No
Prior chemical exposures?	Yes, *if positive:*<br>Specify chemical type and/or setting of exposure<br>No
History of retinoid therapy?	Yes, *if positive:*<br>Type (retinol/al, tretinoin, isotretinoin, other)<br>Form: oral/topical<br>No
Prior head and neck surgery?	Yes, *if positive:*<br>Number of surgeries<br>Type of surgery<br>No
Prior cancer diagnosis?	Yes, *if positive:*<br>Type of Cancer + Treatment modality<br>No
Other cancer diagnosis after UM diagnosis (UM patients only)?	Yes, *if positive:*<br>Type of Cancer<br>No
Does the patient have any family history of cancer?	Yes, *if positive:*<br>Number of family members<br>Relationship to patient<br>Type of cancer<br>No
Does the patient have any family history of cutaneous melanoma?	Yes, *if positive:*<br>Number of family members<br>Relationship to patient<br>No
Does the patient have any family history of ocular melanoma?	Yes, *if positive:*<br>Number of family members<br>Relationship to patient<br>No
Did the patient have any prior eye trauma?	Yes, *if positive:*<br>Laterality (left/right/both)<br>Age of trauma<br>Mechanism of injury<br>No
Did the patient have pre-existing choroidal nevi?	Yes, *if positive:*<br>Laterality (left/right/both)<br>No
Did the patient have pre-existing oculodermal melanocytosis?	Yes, *if positive:*<br>Laterality (left/right/both sides)<br>No
Was the patient pregnant before or at the time of UM diagnosis (for controls, at the time of chart abstraction)?	Yes, recently pregnant<br>Yes, pregnant in the past before the one-year window before UM diagnosis/chart abstraction<br>Record number of pregnancies<br>No<br>N/A, male
